# Dataset of photosynthesis and photosynthetic factors measurements of greenhouse tomato

**DOI:** 10.1016/j.dib.2020.106274

**Published:** 2020-09-03

**Authors:** Jorge Manjarrez-Sanchez, Gerardo Martinez-Carrillo

**Affiliations:** Tecnológico Nacional de México, Mexico

**Keywords:** Greenhouse tomato, Photosynthesis performance, Photosynthesis rate, Tomato physiology

## Abstract

This article presents a set of photosynthetic responses and related environmental variables from greenhouse tomato plants (Lycopersicum esculentum). The dataset was obtained by direct in situ measurement of functional leaves of 100 different plants with a portable photosynthesis system. The measurements were taken for six hours at different time intervals in 16 different days during the cycle of November 2019 to January 2020. This dataset can be used to understand the physiology of greenhouse tomato plants and for the design of photosynthesis forecasting models. It is also useful in precision agriculture and for designing automatic controllers for optimal temperature, humidity, and CO_2_ enrichment, with the purpose of maximizing productivity and avoiding waste of resources. Additionally, this dataset can be used for research in time series analysis.

## Specifications Table

**Table:** 

Subject	Agronomy and Crop Science
Specific subject area	Plant physiology, Photosynthesis
Type of data	Table
How data were acquired	Direct non-destructive leaf measurement with a LI-6400XT Portable Photosynthesis System and OPEN software version 6.2
Data format	Raw
Parameters for data collection	On the field measurements following equipment manufacturer preparation checklist and operating instructions [1].
Description of data collection	In situ leaf enclosed in chamber for measurement of CO_2_ exchange in differential mode.
Data source location	City/Region: Villa de Cos/Zacatecas Country: Mexico Latitude and longitude for collected samples/data: 230 14’ 02” N and 1020 21’ 2” W, 1944 mamsl.
Data accessibility	Repository name: Mendeley Data Data identification number: 10.17632/28zbsynrfm.1 Direct URL to data: https://data.mendeley.com/datasets/28zbsynrfm/1

## Value of the Data

• This dataset can be used to understand the physiology of greenhouse tomato plants. It has values for the climatic and physiological elements involved with the photosynthesis process.
• This dataset is useful for engineers and producers interested in precision agriculture and in the design of automatic controllers.
• The dataset could serve as a sample of the characterization of a natural process for plant bioengineering and synthetic biology.
• Furthermore, these data can be used for the derivation of photosynthesis forecasting models and as a dataset for time series analysis.

## 1 Data Description

The portable photosynthesis meter produces values for the photosynthetic factors and the physiological variables of the leaves related with the photosynthesis process [2], [3], [4], such as the responses of plants to stomatal conductance, reference water in the cell, relative humidity in the cell, transpiration rate, atmospheric pressure, different temperatures in cell space and on plant surfaces.

These measures are provided in data tables separated in two folders:

• 100-1 min – contains files for 100 measures taken in 2 modules, approximately every 1 min, for a total of 200 each day.
• 50–15 min – contains files for 50 daily measures, taken between 5 and 15 minutes approximately.

Each folder contains several excel files with the name Photo*X*M_*date.xls*, where *X* is the number of measures taken and *date* is the corresponding day. Each file has a table, where every row is a measure with 56 columns [1]. Their names and corresponding description are in Table 1.

**Table 1:** Data values.

Value	Description
Obs	# Obs stored in log file
HHMMSS	Real Time Clock
FTime	Time since logging started
EBal?	Do energy Balance?
Photo	Photosynthetic rate
Cond	Conductance to H_2_O
Ci	Intercellular CO_2_ concentration
Trmmol	Transpiration rate
VpdL	Vapor pressure deficit based on Leaf temp
CTleaf	Computed leaf temp (C). Same as Tleaf C unless doing energy balance
Area	In-chamber leaf Area
BLC_1	Not used by this version (Version 6)
StmRat	Stomatal Ratio estimate
BLCond	Not used by this version (Version 6)
Tair	Temperature in sample cell
Tleaf	Temperature of the leaf thermocouple
TBlk	Temperature of cooler block
CO2R	Reference Cell CO_2_
CO2S	Sample Cell CO_2_
H2OR	Reference Cell H_2_O
H2OS	Sample Cell H_2_O
RH_R	Relative Humidity in the reference cell
RH_S	Relative Humidity in the sample cell
Flow	Flow rate to the sample cell
PARi	In-Chamber quantum sensor
PARo	External quantum sensor
Press	Atmospheric Pressure
CsMch	Sample CO_2_ offset umol/mol
HsMch	Sample H_2_O offset umol/mol
StableF	Stability status as a decimal value
BLCslope	Slope as function of area
BLCoffst	Offset as function of area
f_parin	Fraction of ParIn_um to use for EB
f_parout	Fraction of Parout_um to use for EB
alphaK	Adsorption factor alpha*conversion factor k
Status	Numerical status code
fda	flow / area with unit conversion
Trans	Transpiration
Tair_K	Chamber Air Temperature
Twall_K	Wall Temperature
R(W/m2)	Incoming Radiation
Tl-Ta	Energy Balance Delta T
SVTleaf	SatVap(Tleaf)
h2o_i	Intercellular H2O
h20diff	Diff
CTair	Air Temperature in the leaf chamber
SVTair	SatVap(air)
CndTotal	Total Conductance
vp_kPa	Vapor pressure chamber air
VpdA	Vapor pressure deficit based on Air Temp
CndCO2	Total Conductance to CO_2_
Ci_Pa	Intercellular CO_2_
Ci/Ca	Intercellular CO_2_/Ambient CO_2_
RHsfc	Surface Humidity
C2sfc	Surface CO_2_
AHs/Cs	Ball-Berry Parameter

## 2 Experimental Design, Materials and Methods

The researched crop is greenhouse tomato (Lycopersicum esculentum). It was grown in a hydroponic system of coconut substrate in bags of 90 cm long and 28 l with 3 plants each, with a density of 2.6 plants/m^2^. The Steiner nutrient solution that was used in the emitter of the drip irrigation system, had an electrical conductivity of 2.5 dS m^−1^. The greenhouse structure has 50 m long, 10 m width and 5 m height with a north-south orientation in the direction of the gutters.

The dataset was obtained in 16 different days in the cycle of November 2019 to January 2020, by direct non-destructive measuring of carbon dioxide exchange of a functional leaf. The measures were obtained with a portable photosynthesis system LI-6400XT with OPEN software version 6.2 [1]. During the experiment, the air flow was constant at 400 µmol s^−^^1^ and the CO_2_ supply at 400 ppm. The temperature of the device chamber was not maintained constant. The values for the photosynthetic rate, intercellular CO_2_ concentration, photosynthetically active radiation (PAR) and, temperature of the leaf were obtained in 2 different sets: the first one contains measurements taken approximately every minute from 100 plants in two modules, for a total of 200 measurements each day. The second set is for measurements taken every 5–15 min from about 50 plants.

## Declaration of Competing Interest

The authors declare that they have no known competing financial interests or personal relationships which have, or could be perceived to have, influenced the work reported in this article.

## Appendix Supplementary materials

Image, application 1
